# HBV-Specific Adaptive Immunity

**DOI:** 10.3390/v1020091

**Published:** 2009-07-27

**Authors:** Antonio Bertoletti, Anthony T. Tan, Adam J. Gehring

**Affiliations:** 1 Laboratory of Hepatic Viral Diseases, Singapore Institute for Clinical Sciences, Agency of Science Technology and Research (A*Star), 30 Medical Drive, 117609, Singapore; E-Mails: t.a.tanoto@gmail.com; adam_gehring@sics.a-star.edu.sg; 2 Singapore Immunology Network, Agency of Science Technology and Research (A*Star), Singapore; 3 Program Emerging Viral Diseases Unit, Duke-NUS Graduate Medical School, Singapore

**Keywords:** HBV, T cells, B cells

## Abstract

The successful control of HBV infection requires an efficient expansion of distinct elements of the adaptive immune system (B cells, helper and cytotoxic T cells) that, due to the hepatotropic nature of HBV, need to operate in the liver parenchyma. In this respect, we will discuss broad features of HBV immunity in patients with resolved or chronic HBV infection and analyze how the liver environment can directly modulate HBV-immunity.

## Introduction

**Figure 1.**
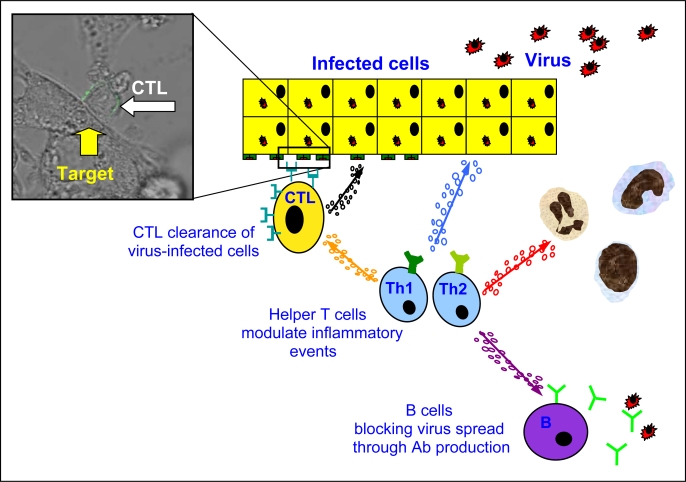
Anti-viral adaptive immune response during HBV infection.

A network of cell types, which can play protective or pathogenic roles, forms the anti-viral adaptive immune response during HBV infection. These different components are: a) CD4 T cells, classically referred to as helper T cells. They are robust producers of cytokines and are required for the efficient development of effector cytotoxic CD8 T cells and B cell antibody production. In addition, CD4 T cells can potentially modulate inflammatory events through secretion of pro (CXCL-8, IL-17) or anti-inflammatory (IL-10) cytokines. b) CD8 T cells are able to directly recognize virus-infected cells and clear HBV-infected hepatocytes through cytolytic and non-cytolytic mechanisms [1, 2], reducing the levels of circulating virus. c) B cells can neutralize free viral particles through antibody production to prevent (re)infection [3, 4] and might modulate helper T cell function through their ability to present HBV antigens (Figure 1).

The ability to develop and preserve an efficient HBV-specific adaptive immune cell network is thought to represent the most important discriminatory factor between HBV control or chronicity [5, 6]. Such ability is influenced by events occurring in the initial phases of infection and is related to the innate immune response, which will not be covered in this review and has already been extensively discussed [7, 8]. In this review we will briefly summarize the different features of adaptive immunity in resolved versus chronic patients and the potential causes of the apparent collapse of HBV-immunity during chronicity. We will also discuss different variables that can alter virus-specific T cell function in the intrahepatic compartment.

## HBV-adaptive immunity: control versus chronicity

What is clear in HBV infection is that HBV-specific CD4 and CD8 T cell frequency and function is far superior in patients who resolve HBV infection than in subjects with chronic infection. The ability of patients who resolve the infection to produce anti-viral cytokines (IFN-γ and TNF-α) results in HBV clearance from infected hepatocytes without extensive direct killing [9–20]. The contribution of B cells and antibody are also important in HBV control, although these components of adaptive immunity have attracted less scientific attention in comparison to T cells. Nevertheless, HBV clearance is associated with the production of anti-envelope antibodies [3], and sera with high levels of anti-viral antibodies (specific for the viral envelope) can control HBV infection [4].

A level of coordination between the different components of adaptive immunity seems necessary to achieve sustained HBV control. For example, woodchucks infected with WHV (woodchuck hepatitis B virus) demonstrate the importance of a coordinated helper and cytotoxic T cell response in controlling hepadnavirus infections. It was observed that a reduced early expansion of virus-specific T cells during WHBV infection was associated with virus persistence [21]. In patients studied during the incubation phase of acute HBV infections, expansion of virus-specific IFN-γ+ CD8 and CD4 T cells preceded complete virus clearance and this response was present only in subjects who controlled the infection [22]. Furthermore, a recent analysis of HBV-specific T cell responses in an HBV-HCV acutely co-infected patient, who developed a persistent HBV infection, showed the presence of a multi-specific CD8 T cell response in the absence of a CD4 T cell response [23]. It is likely that the absence of CD4 help prevented the maturation of a functionally effective CD8 T cell response.

Interestingly, the development of functional HBV-specific memory CD8+ T cells is linked to down-regulation of PD-1 expression (and up-regulation of the IL-7 receptor CD127) on HBV-specific CD8 cells [24, 25]. Increased functional activity of the HBV-specific CD8+ T cell response during the late stages of acute HBV infection has been previously reported [16], but these new data establish a direct link between over-expression of PD-1, functional alteration, and acquisition of functional efficiency. We will discuss in the next paragraph how, instead, prolonged over-expression of PD-1 constitutes a hallmark of the functionally altered T cells present during persistent HBV infection.

Since most of these studies have been performed in humans, their observational nature does not allow us to firmly assert that T-cell defects observed in chronic HBV patients are the cause or the consequence of HBV persistence. However, studies in animal models and clinical observations are filling this gap. CD8 T cell deletion experiments performed in HBV-infected chimpanzees have provided strong support for the concept that CD8 T cells are responsible for viral clearance [26]. Furthermore, chronic HBV patients who underwent bone marrow transplantation and received marrow from donors with natural immunity to HBV resolved chronic HBV infection [27, 28, 29]. Similarly, a recent report showed HBV clearance from a HBV chronically infected liver that was transplanted into a subject with a prior self-limited HBV infection [30]. In both examples, HBV clearance from the chronically infected liver was, as in the natural self-limited HBV infection [9, 10], associated with an HBV-specific T-cell response and production of anti-envelope antibodies. Taken together, these examples suggest that it may be a weak or misdirected immune response that leads to chronic HBV infection rather than any property of the virus itself. Reconstitution of the immune system by bone marrow transplant or transplanting chronically infected liver into a patient with HBV immunity achieved viral clearance [27–30], suggesting that there was nothing inherently persistent in the virus infecting these patients.

Additional evidence that CD4 and CD8 T-cell responses are accountable for the immunological control of HBV are represented by the association of particular HLA class I and class II genetic profiles with resolution [31]. Interestingly, HLA class II heterozygosis is associated with HBV clearance, supporting the hypothesis that multispecific HBV-specific HLA-class II-restricted T cells can protect from HBV persistence [32].

## Adaptive immunity in chronic HBV infection

Chronic viral infection alters the quantitative and functional profile of virus-specific immunity. Detailed functional studies have mainly focused on the analysis of CD8 T cells, which during persistent viral infection lose their ability to lyse, proliferate and produce different cytokines (i.e. TNF-alpha and IL-2), and only partially maintain the ability to produce IFN-gamma [33]. This dysfunctional state, or exhaustion, is characterized by CD8 T cell over-expression of inhibitory receptors (PD-1, Lag-3 and CTLA-4), major changes in T-cell receptor and cytokine signaling pathways, and altered expression of genes involved in adhesion and migration. In addition, exhausted virus-specific T cells show profound metabolic and bio-energetic deficiencies [34].

HBV-specific CD8+ T cells detected during chronic infection often display similar defects. They produce low levels of antiviral cytokines, proliferate poorly in vitro, express the inhibitory receptor PD-1, and are prone to apoptosis [20, 35]. However, dysfunctional HBV-specific T cells are not the classical hallmark of patients with chronic HBV infection. In reality, such a dysfunctional population is mainly detectable in the blood of chronic HBV patients with low level of HBV-replication (around < 0.5–1 × 10^6^ HBV-DNA IU/mL) and HBV-specific T cells are often undetectable in the majority of patients with higher levels of HBV replication (HBeAg+, HBV-DNA IU/mL > 0.5–1 × 10^8^). T cells specific for HBV antigens that are more expressed in the liver are deleted in HBV transgenic mice [36], and the hierarchy of epitope-specific CD8+ T cells is altered in proportion to the level of HBV DNA in chronically infected patients. For example, CD8 T cells, specific for a dominant core epitope, cannot be found in the circulation or liver when HBV viremia exceeds 0.5–1 × 10^7^ copies/mL [18]. Importantly, the deletion/alteration of HBV-specific cells is not limited to the cellular arm of immunity, but also affects the humoral response, as HBV-specific antibody production is clearly impaired in chronic patients [37].

We believe that the prolonged exposure to large quantities of soluble HBV antigens (HBsAg and HBeAg) and the tolerogenic features of the liver are the principal causes of the functional alteration and deletion of virus-specific B and T cells present in chronic HBV patients. Antigen persistence alters T-cell function and causes deletion in different viral infections [38]. In HBV infection, the immune system has to handle unusually high quantities of viral antigens. HBeAg, a secretory form of the HBV nucleocapsid, or core antigen, is produced during HBV replication. The tolerizing effect of HBeAg has been well characterized in mice and is likely contributing to the low level of core-specific T-cell responses present in chronic HBV patients. HBsAg is also produced in large excess during HBV infection. Particles composed of only HBsAg are produced in 10^3^–10^6^ fold excess over whole virions and can reach 1–10 ug/mL in the serum [39].

Such a high and persistent load of viral antigens allows them to be cross-presented by liver professional antigen-presenting cells (Kuppfer cells, liver endothelial cells), which can have a tolerizing effect [40, 41]. This might well explain the level of the T cell hyporesponsiveness present in HBV chronic patients. In addition, direct presentation of foreign antigen by hepatocytes has been suggested to preferentially induce tolerance in CD8 T cells, reducing T cell expansion and promoting T cell apoptosis [42–44]. However, this scenario is still controversial since other work in mice has shown rapid activation and expansion of naïve and effector CD8 T cells following hepatocyte presentation of viral antigen [45].

Additional factors might contribute to the maintenance, if not directly induce, HBV-specific immune dysfunction. Some could exert their function mainly in the intrahepatic environment, while others are not topologically restricted to the liver (Table 1). Regarding the latter, we include dendritic cell functional alteration and T regulatory cells. Dendritic cells represent a specialized antigen-presenting cell population necessary for the efficient induction of an adaptive immune response [46]. We think that the evidence for an impact of altered dendritic cell function in causing and/or maintaining the HBV-specific immune defects during chronic HBV infection is fragile. Dendritic cells can be infected in animal models of hepadnavirus infection [47], but productive HBV replication in dendritic cells has recently been excluded in chronic hepatitis B patients [48, 49], and the stimulatory defects observed seem minimal [50–53]. A recent report described an up-regulation of PD-L1 on myeloid dendritic cells of chronic HBV patients, suggesting a generalized T cell inhibitory effect of the PD-1/PD-L1 interaction mediated by dendritic cells [54]. These data would imply a global impairment of the immune system which is not evident from a study performed on a large group of chronic HBV patients of different ethnicities [55].

The impact of T regulatory cells in the modulation of HBV-specific T cell immunity is also controversial (see also [56, 57]). Treg cells are diverse, occurring in both CD4 and CD8 T cell subsets. They express various markers such as CD25 (IL-2 receptor α chain), cytotoxic T-lymphocyte antigen 4 (CTLA-4) and the forkhead family transcription factor (FoxP3) [58], and can potentially suppress anti-HBV immunity through cell to cell contact or production of regulatory cytokines like IL-10 [59]. Studies have reported an increased frequency of circulating regulatory cells in all patients with chronic hepatitis B [60], but others either found that this quantitative correlation was only present in patients with severe disease or could not confirm such data [61, 62]. Similar inconsistencies were detected when Treg cell frequency was analyzed in relation to viral load [57] and when the functionality of Treg cells was examined. Depletion of CD4+CD25+ cells increased the function of HBV-specific T cells [62, 63, 64] *in vitro*, but such modulation was HBV-specific for some [63], but not others [62].

We think it is important to consider that these studies were limited to the analysis of the CD4+ CD25+ cells present in the blood, while a detailed analysis of the intrahepatic frequency and function of these cells has not been performed. Furthermore, it is possible that a population of HBV-specific regulatory cells, different from the CD4+ CD25+ T cell subset, analogous to the presence of IL-10-producing HCV-specific T cells [65], might be present in chronic HBV infection. There have been initial reports of the presence of such HBV-specific IL-10-producing Treg in patients with chronic hepatitis [66, 67], but the frequency of such cells appears extremely low and could not been confirmed in more recent analysis (Gehring, A.; Bertoletti, A. Manuscript in preparation).

## T cell response and function within the liver

We have discussed the features of adaptive immunity in patients with chronic and resolved HBV infection and the potential mechanisms that are likely to affect their quantitative and qualitative profile. A final issue that we would like to briefly discuss is the modulation of effector T cell function caused by the liver environment. This aspect is different from the tolerogenic features of the liver which act on the induction phase, and instead deals with the potential direct influence of liver environment and hepatocyte antigen presentation on T cell effector function.

The fact that virally infected hepatocytes are largely resistant to perforin/granzyme-mediated killing [68], but highly sensitive to cytokine-mediated clearance of HBV [69], illustrates how T cell effects can be different in the liver environment. However, new aspects such as modulation of T cell function induced by hepatocyte antigen presentation [70], metabolism in the liver environment with the ability of Arginase to alter CD3 zeta expression on T cells [71], and the interaction with other cell types (i.e. platelets) [72, 73], have recently been reported in the scientific literature.

Isogawa *et al.* have shown in an *in vivo* model of HBV infection that effector T cells can change their cytolytic or non-cytolytic function after recognition of antigen presented by hepatocytes [70]. Their data show oscillation of effector T cell function, which mainly produce cytokines when initially triggered by a high dose of HBV antigens and then switch their function toward a restricted cytolytic effect. This functional swing might be regulated by the expression of PD-1 on effector cells [74] or be a direct consequence of antigen dose [70]. Both hypotheses are in agreement with human data. PD1 and PD-L1 interaction can occur in the liver, since PD-L1 is expressed by infected hepatocytes [75]. On the other hand, recent analyses performed in vitro using human CTL clones and T cell effector lines have shown that the quantity of viral antigen presented by hepatocytes can influence CD8 T cell anti-viral function. High levels of HBV production induced robust IFN-γ production in virus-specific CD8 T cells, while limiting amounts of viral antigen, both in hepatocyte-like cells and naturally infected human hepatocytes, preferentially stimulated CD8 T cell degranulation [76].

The possibility that increased availability of MHC-viral peptide complexes on hepatocytes, as a consequence of different levels of HBV replication, could trigger differential function in HBV-specific T cells is consistent with clinical and new experimental observations. Hepatic flares are associated with increased levels of HBV replication (> 10^7^ copies/mL serum), while chronic patients that control HBV replication (< 10^6^ copies/mL serum) can do so without signs of liver inflammation. In both situations, the quantity of intrahepatic HBV-specific CD8+ T cells are similar and the discriminatory factor is the level of HBV replication, suggesting that the availability of antigen modulates T cell function [76].

The concept that effector T cells can modulate their function in relation to different levels of hepatocyte antigen presentation is further supported by recent observations that classical Th1/Tc1 HBV-specific T cells can produce the neutrophil chemotactic factor CXCL-8 when stimulated by high antigen dose. Parenchymal recruitment of granulocytes is a common step of immunopathological processes triggered by CTLs during infection with non-cytopathic viruses [77], and seems particularly important in the liver, where intra-hepatic neutrophil activation digests collagen and opens the liver parenchyma for inflammatory mononuclear cell infiltration [78, 79].

The cross-talk between different components of immunity, like the ability of HBV-specific T cells to produce chemokines involved in the recruitment of granulocytes, opens a further chapter; the influence of other cell types on the function of adaptive immunity in the liver. Work on HBV transgenic mice has clearly shown that activated platelets influence the recruitment of virus-specific CTLs into the liver [72]. Platelet depletion reduced intrahepatic accumulation of virus-specific CTLs and liver damage without impairing the effector functions of CTLs. Such results were obtained both in HBV transgenic [80] and LCMV-infected mice [73]. The specific interaction between activated platelets and virus-specific CTLs seems to favor their accumulation at the site of inflammation, a process mediated by serotonin.

Thus, protective or pathogenic effects of HBV-specific T cells in the liver are not only dependent on their quantity and intrinsic function, but can be modulated by external factors like platelets and granulocytes. In addition to these data, a recent report by Das *et al*. has shown that depletion of arginine in the inflamed liver can directly interfere with intrahepatic T cell function, particularly with their ability to produce IL-2 and proliferate [71]. This adds a further layer of complexity in T cell effector function in an HBV-infected liver and the balance between control of HBV infection or chronicity.

## Concluding remarks

Beyond the well characterized differences in HBV adaptive immunity of resolved and chronic patients lie a number of issues that have been difficult to determine. We know that the host response to HBV infection is a complex coordinated process and that the liver environment might tune effector T cell function. Studies have begun to shed light on the peculiarity of the intrahepatic environment and how this can modulate virus-specific immunity. In fact, modification of the liver environment may already be the basis of the therapeutic effect of IFN-alpha that, through activation of the immunoproteasomes in hepatocytes, can change the quantity of HLA-class I/peptide complexes available for CD8+ T cell recognition. These types of questions are exceedingly difficult to study in humans but it is necessary to understand how the immune system operates in the liver if immunotherapeutic strategies under development are to have any success in achieving sustained viral control.

In addition to the liver environment, a better understanding of how the network of immune cells, parenchymal/non-parenchymal cells, and platelets interact and communicate to achieve HBV clearance or liver damage is critical. Multiple components are involved in each process and being able to separate protective from inflammatory processes could increase the efficacy and safety of HBV treatment. As a further and final note, the study of HBV-specific adaptive immunity in patients is heavily skewed towards analysis of T cell function. Although there is no doubt that T cells play an essential role during HBV infection, perhaps new exciting revelations of HBV pathogenesis will derive from new accurate studies of HBV-specific B cell function.

## Figures and Tables

**Table 1.** Mechanisms regulating function of HBV-specific T cell immunity.

***Broad - Not organ specific***	***Liver specific***

High dose of antigen-	Metabolic feature of the liver (Arginase)
Reduced pro-inflammatory cytokine production.	Hepatocytes presentation (inhibitory molecules / HLA-class I)
Dendritic cells impairment / T regulatory cells	Interaction with other cells (i.e.Platelets-granulocytes)

## References

[1] Chisari F (1997). Cytotoxic T cells and viral hepatitis. J Clin Invest.

[2] Guidotti L, Chisari F (1996). To kill or to cure: options in host defense against viral infection. Current Opin Immunol.

[3] Alberti A, Diana S, Sculard GH, Eddleston AL, Williams R (1978). Detection of a new antibody system reacting with Dane particles in hepatitis B virus infection. Br Med J.

[4] Grady GF, Lee VA, Prince AM, Gitnick GL, Fawaz KA, Vyas GN, Levitt MD, Senior JR, Galambos JT, Bynum TE, Singleton JW, Clowdus BF, Akdamar K, Aach RD, Winkelman EI, Schiff GM, Hersh T (1978). Hepatitis B immune globulin for accidental exposures among medical personnel: final report of a multicenter controlled trial. J Infect Dis.

[5] Chisari F, Ferrari C (1995). Hepatitis B virus immunopathogenesis. Ann Rev Immunol.

[6] Bertoletti A, Gehring AJ (2006). The immune response during hepatitis B virus infection. J Gen Virol.

[7] Fisicaro P, Valdatta C, Boni C, Massari M, Mori C, Zerbini A, Orlandini A, Sacchelli L, Missale G, Ferrari C (2009). Early kinetics of innate and adaptive immune responses during hepatitis B virus infection. Gut.

[8] Wieland SF, Chisari FV (2005). Stealth and cunning: hepatitis B and hepatitis C viruses. J Virol.

[9] Ferrari C, Penna A, Bertoletti A, Valli A, Degli Antoni A, Giuberti T, Cavalli A, Petit MA, Fiaccadori F (1990). Cellular immune response to hepatitis B virus encoded antigens in acute and chronic hepatitis B virus infection. J Immunol.

[10] Jung M, Spengler U, Schraut W, Hoffman R, Zachoval R, Eisemburg J, Eichenlaub D, Riethmuller G, Paumgartner G, Ziegler-Heitbrock HWL, Pape GR (1991). Hepatitis B virus antigen-specific T-cell activation in patients with acute and chronic hepatitis B. J Hepatol.

[11] Penna A, Artini M, Cavalli A, Levrero M, Bertoletti A, Pilli M, Chisari FV, Rehermann B, Del Prete G, Fiaccadori F, Ferrari C (1996). Long-lasting memory T cell responses following self-limited acute hepatitis B. J Clin Invest.

[12] Penna A, Del Prete G, Cavalli A, Bertoletti A, D'Elios MM, Sorrentino R, D'Amato M, Boni C, Pilli M, Fiaccadori F, Ferrari C (1997). Predominant T-helper 1 cytokine profile of hepatitis B virus nucleocapsid-specific T cells in acute self-limited hepatitis B. Hepatology.

[13] Rehermann B, Fowler P, Sidney J, Person J, Redeker A, Brown M, Moss B, Sette A, Chisari FV (1995). The cytotoxic T lymphocyte response to multiple hepatitis B virus polymerase epitopes during and after acute viral hepatitis. J Exp Med.

[14] Penna A, Chisari FV, Bertoletti A, Missale G, Fowler P, Giuberti T, Fiaccadori F, Ferrari C (1991). Cytotoxic T lymphocytes recognize an HLA-A2-restricted epitope within the hepatitis B virus nucleocapsid antigen. J Exp Med.

[15] Jung M, Hartmann B, Gerlach J, Diepolder H, Gruber R, Schraut W, Gruner N, Zachoval R, Hoffmann R, Santantonio T, Pape G (1999). Virus-specific lymphokine production differs quantitatively but not qualitatively in acute and chronic hepatitis B infection. Virology.

[16] Maini MK, Boni C, Ogg GS, King AS, Reignat S, Lee CK, Larrubia JR, Webster GJM, McMichael AJ, Ferrari C, Williams R, Vergani D, Bertoletti A (1999). Direct ex vivo analysis of hepatitis B virus-specific CD8+ T cells associated with the control of infection. Gastroenterology.

[17] Sobao Y, Tomiyama H, Sugi K, Tokunaga M, Ueno T, Saito S, Fujiyama S, Morimoto M, Tanaka K, Takiguchi M (2002). The role of hepatitis B virus-specific memory CD8 T cells in the control of viral replication. J Hepatol.

[18] Webster GJ, Reignat S, Brown D, Ogg GS, Jones L, Seneviratne SL, Williams R, Dusheiko G, Bertoletti A (2004). Longitudinal analysis of CD8+ T cells specific for structural and nonstructural hepatitis B virus proteins in patients with chronic hepatitis B: implications for immunotherapy. J Virol.

[19] Chang JJ, Wightman F, Bartholomeusz A, Ayres A, Kent SJ, Sasadeusz J, Lewin SR (2005). Reduced hepatitis B virus (HBV)-specific CD4+ T-cell responses in human immunodeficiency virus type 1-HBV-coinfected individuals receiving HBV-active antiretroviral therapy. J Virol.

[20] Boni C, Fisicaro P, Valdatta C, Amadei B, Di Vincenzo P, Giuberti T, Laccabue D, Zerbini A, Cavalli A, Missale G, Bertoletti A, Ferrari C (2007). Characterization of hepatitis B virus (HBV)-specific T-cell dysfunction in chronic HBV infection. J Virol.

[21] Menne S, Roneker CA, Roggendorf M, Gerin JL, Cote PJ, Tennant BC (2002). Deficiencies in the acute-phase cell-mediated immune response to viral antigens are associated with development of chronic woodchuck hepatitis virus infection following neonatal inoculation. J Virol.

[22] Webster G, Reignat S, Maini M, Whalley S, Ogg G, King A, Brown D, Amlot P, Williams R, Vergani D, Dusheiko G, Bertoletti A (2000). Incubation phase of acute Hepatitis B in man: dynamic of cellular immune mechanism. Hepatology.

[23] Urbani S, Boni C, Amadei B, Fisicaro P, Cerioni S, Valli MA, Missale G, Ferrari C (2005). Acute phase HBV-specific T cell responses associated with HBV persistence after HBV/HCV coinfection. Hepatology.

[24] Boettler T, Panther E, Bengsch B, Nazarova N, Spangenberg HC, Blum HE, Thimme R (2006). Expression of the interleukin-7 receptor alpha chain (CD127) on virus-specific CD8+ T cells identifies functionally and phenotypically defined memory T cells during acute resolving hepatitis B virus infection. J Virol.

[25] Zhang Z, Jin B, Zhang JY, Xu B, Wang H, Shi M, Wherry EJ, Lau GK, Wang FS (2009). Dynamic decrease in PD-1 expression correlates with HBV-specific memory CD8 T-cell development in acute self-limited hepatitis B patients. J Hepatol.

[26] Thimme R, Wieland S, Steiger C, Ghrayeb J, Reimann KA, Purcell RH, Chisari FV (2003). CD8(+) T cells mediate viral clearance and disease pathogenesis during acute hepatitis B virus infection. J Virol.

[27] Ilan Y, Nagler A, Adler R, Tur-Kaspa R, Slavin S, Shouval D (1993). Ablation of persistent hepatitis B by bone marrow transplantation from a hepatitis B-immune donor. Gastroenterology.

[28] Lau GK, Lok AS, Liang RH, Lai CL, Chiu EK, Lau YL, Lam SK (1997). Clearance of hepatitis B surface antigen after bone marrow transplantation: role of adoptive immunity transfer. Hepatology.

[29] Lau GK, Suri D, Liang R, Rigopoulou EI, Thomas MG, Mullerova I, Nanji A, Yuen ST, Williams R, Naoumov NV (2002). Resolution of chronic hepatitis B and anti-HBs seroconversion in humans by adoptive transfer of immunity to hepatitis B core antigen. Gastroenterology.

[30] Loggi E, Bihl F, Chisholm JV, Biselli M, Bontadini A, Vitale G, Ercolani G, Grazi G, Pinna AD, Bernardi M, Brander C, Andreone P (2009). Anti-HBs re-seroconversion after liver transplantation in a patient with past HBV infection receiving a HBsAg positive graft. J Hepatol.

[31] Thio CL, Thomas DL, Karacki P, Gao X, Marti D, Kaslow RA, Goedert JJ, Hilgartner M, Strathdee SA, Duggal P, O'Brien SJ, Astemborski J, Carrington M (2003). Comprehensive analysis of class I and class II HLA antigens and chronic hepatitis B virus infection. J Virol.

[32] Thursz M, Thomas H, Greenwood B, Hill A (1997). Heterozygote advantage for HLA-class II type in hepatitis B virus infection. Nature Genet.

[33] Wherry EJ, Blattman JN, Murali-Krishna K, van der Most R, Ahmed R (2003). Viral persistence alters CD8 T-cell immunodominance and tissue distribution and results in distinct stages of functional impairment. J Virol.

[34] Wherry EJ, Ha SJ, Kaech SM, Haining WN, Sarkar S, Kalia V, Subramaniam S, Blattman JN, Barber DL, Ahmed R (2007). Molecular Signature of CD8+ T Cell Exhaustion during Chronic Viral Infection. Immunity.

[35] Lopes AR, Kellam P, Das A, Dunn C, Kwan A, Turner J, Peppa D, Gilson RJ, Gehring A, Bertoletti A, Maini MK (2008). Bim-mediated deletion of antigen-specific CD8 T cells in patients unable to control HBV infection. J Clin Invest.

[36] Kakimi K, Isogawa M, Chung J, Sette A, Chisari FV (2002). Immunogenicity and tolerogenicity of hepatitis B virus structural and nonstructural proteins: implications for immunotherapy of persistent viral infections. J Virol.

[37] Maruyama T, McLachlan A, Iino S, Koike K, Kurokawa K, Milich D (1993). The serology of chronic hepatitis B infection revisited. J Clin Invest.

[38] Welsh R (2001). Assessing CD8 T cell number and dysfunction in the presence of antigen. J Exp Med.

[39] Ganem D, Prince AM (2004). Hepatitis B virus infection—natural history and clinical consequences. N Engl J Med.

[40] Limmer A, Ohl J, Kurts C, Ljunggren H-G, Reiss Y, Groettrup M, Momburg F, Arnold B, Knolle P (2000). Efficient presentation of exogenous antigen by liver endothelial cells to CD8+ T cells results in antigen-specific T cell tolerance. Nature Med.

[41] Crispe IN (2003). Hepatic T cells and liver tolerance. Nat Rev Immunol.

[42] Bertolino P, Bowen D, McCaughan G, Fazekas de St Groth B (2001). Antigen-specific primary activation of CD8+ T cells within the liver. J Immunol.

[43] Bertolino P, Trescol-Biemont M-C, Rabourdin-Combe C (1998). Hepatocytes induce functional activation of naive CD8+ T lymphocytes but fail to promote survival. Eur J Immunol.

[44] Bowen DG, Zen M, Holz L, Davis T, McCaughan GW, Bertolino P (2004). The site of primary T cell activation is a determinant of the balance between intrahepatic tolerance and immunity. J Clin Invest.

[45] Murray DA, Crispe IN (2004). TNF-alpha controls intrahepatic T cell apoptosis and peripheral T cell numbers. J Immunol.

[46] Banchereau J, Briere F, Caux C, Davoust J, Lebecque S, Liu YJ, Pulendran B, Palucka K (2000). Immunobiology of dendritic cells. Annu Rev Immunol.

[47] Lew YY, Michalak TI (2001). In vitro and in vivo infectivity and pathogenicity of the lymphoid cell-derived woodchuck hepatitis virus. J Virol.

[48] Tavakoli S, Schwerin W, Rohwer A, Hoffmann S, Weyer S, Weth R, Meisel H, Diepolder H, Geissler M, Galle PR, Lohr HF, Bocher WO (2004). Phenotype and function of monocyte derived dendritic cells in chronic hepatitis B virus infection. J Gen Virol.

[49] Untergasser A, Zedler U, Langenkamp A, Hosel M, Quasdorff M, Esser K, Dienes HP, Tappertzhofen B, Kolanus W, Protzer U (2006). Dendritic cells take up viral antigens but do not support the early steps of hepatitis B virus infection. Hepatology.

[50] Wang FS, Xing LH, Liu MX, Zhu CL, Liu HG, Wang HF, Lei ZY (2001). Dysfunction of peripheral blood dendritic cells from patients with chronic hepatitis B virus infection. World J Gastroenterol.

[51] Beckebaum S, Cicinnati VR, Dworacki G, Muller-Berghaus J, Stolz D, Harnaha J, Whiteside TL, Thomson AW, Lu L, Fung JJ, Bonham CA (2002). Reduction in the circulating pDC1/pDC2 ratio and impaired function of ex vivo-generated DC1 in chronic hepatitis B infection. Clin Immunol.

[52] Lohr HF, Pingel S, Bocher WO, Bernhard H, Herzog-Hauff S, Rose-John S (2002). Reduced virus specific T helper cell induction by autologous dendritic cells in patients with chronic hepatitis B - restoration by exogenous interleukin-12. Clin Exp Immunol.

[53] van der Molen RG, Sprengers D, Binda RS, de Jong EC, Niesters HG, Kusters JG, Kwekkeboom J, Janssen HL (2004). Functional impairment of myeloid and plasmacytoid dendritic cells of patients with chronic hepatitis B. Hepatology.

[54] Chen L, Zhang Z, Chen W, Zhang Z, Li Y, Shi M, Zhang J, Chen L, Wang S, Wang FS (2007). B7-H1 up-regulation on myeloid dendritic cells significantly suppresses T cell immune function in patients with chronic hepatitis B. J Immunol.

[55] Tan AT, Loggi E, Boni C, Chia A, Gehring AJ, Sastry KS, Goh V, Fisicaro P, Andreone P, Brander C, Lim SG, Ferrari C, Bihl F, Bertoletti A (2008). Host ethnicity and virus genotype shape the hepatitis B virus-specific T-cell repertoire. J Virol.

[56] Bauer T, Gunther M, Bienzle U, Neuhaus R, Jilg W (2007). Vaccination against hepatitis B in liver transplant recipients: pilot analysis of cellular immune response shows evidence of HBsAg-specific regulatory T cells. Liver Transpl.

[57] Manigold T, Racanelli V (2007). T-cell regulation by CD4 regulatory T cells during hepatitis B and C virus infections: facts and controversies. Lancet Infect Dis.

[58] Shevach EM (2006). From vanilla to 28 flavors: multiple varieties of T regulatory cells. Immunity.

[59] van Driel I, Ang D (2008). The role of regulatory T cells in gastrointestinal inflammatory disease. J Gastroenterol Hepatol.

[60] Yang G, Liu A, Xie Q, Guo TB, Wan B, Zhou B, Zhang JZ (2007). Association of CD4+CD25+Foxp3+ regulatory T cells with chronic activity and viral clearance in patients with hepatitis B. Int Immunol.

[61] Kondo Y, Kobayashi K, Ueno Y, Shiina M, Niitsuma H, Kanno N, Kobayashi T, Shimosegawa T (2006). Mechanism of T cell hyporesponsiveness to HBcAg is associated with regulatory T cells in chronic hepatitis B. World J Gastroenterol.

[62] Franzese O, Kennedy PT, Gehring AJ, Gotto J, Williams R, Maini MK, Bertoletti A (2005). Modulation of the CD8+-T-cell response by CD4+ CD25+ regulatory T cells in patients with hepatitis B virus infection. J Virol.

[63] Stoop JN, van der Molen RG, Baan CC, van der Laan LJ, Kuipers EJ, Kusters JG, Janssen HL (2005). Regulatory T cells contribute to the impaired immune response in patients with chronic hepatitis B virus infection. Hepatology.

[64] Xu D, Fu J, Jin L, Zhang H, Zhou C, Zou Z, Zhao JM, Zhang B, Shi M, Ding X, Tang Z, Fu YX, Wang FS (2006). Circulating and liver resident CD4+CD25+ regulatory T cells actively influence the antiviral immune response and disease progression in patients with hepatitis B. J Immunol.

[65] Accapezzato D, Francavilla V, Paroli M, Casciaro M, Chircu LV, Cividini A, Abrignani S, Mondelli MU, Barnaba V (2004). Hepatic expansion of a virus-specific regulatory CD8(+) T cell population in chronic hepatitis C virus infection. J Clin Invest.

[66] Hyodo N, Nakamura I, Imawari M (2004). Hepatitis B core antigen stimulates interleukin-10 secretion by both T cells and monocytes from peripheral blood of patients with chronic hepatitis B virus infection. Clin Exp Immunol.

[67] Chang JJ, Thompson AJ, Visvanathan K, Kent SJ, Cameron PU, Wightman F, Desmond P, Locarnini SA, Lewin SR (2007). The phenotype of hepatitis B virus-specific T cells differ in the liver and blood in chronic hepatitis B virus infection. Hepatology.

[68] Kafrouni MI, Brown GR, Thiele DL (2001). Virally infected hepatocytes are resistant to perforin-dependent CTL effector mechanisms. J Immunol.

[69] Guidotti L, Chisari F (1999). Cytokine-induced viral purging-role in viral pathogenesis. Curr Opin Microbiol.

[70] Isogawa M, Furuichi Y, Chisari FV (2005). Oscillating CD8(+) T cell effector functions after antigen recognition in the liver. Immunity.

[71] Das A, Hoare M, Davies N, Lopes AR, Dunn C, Kennedy PT, Alexander G, Finney H, Lawson A, Plunkett FJ, Bertoletti A, Akbar AN, Maini MK (2008). Functional skewing of the global CD8 T cell population in chronic hepatitis B virus infection. J Exp Med.

[72] Iannacone M, Sitia G, Ruggeri ZM, Guidotti LG (2007). HBV pathogenesis in animal models: recent advances on the role of platelets. J Hepatol.

[73] Lang P, Contaldo C, Georgiev P, El-Badry A, Recher M, Kurren M, Cervantes-Barragan L, Ludewig B, Calzascia T, Bolinger B, Merkler D, Odermatt B, Bader M, Graf R, Clavien PA, Hegazy A, Lohning M, Harris N, Ohashi PS, Hengartner H, Zinkernagel RM, Lang KS (2008). Aggravation of viral hepatitis by platelet-derived serotonin. Nat Med.

[74] Maier H, Isogawa M, Freeman GJ, Chisari FV (2007). PD-1:PD-L1 interactions contribute to the functional suppression of virus-specific CD8+ T lymphocytes in the liver. J Immunol.

[75] Mühlbauer M, Fleck M, Schütz C, Weiss T, Froh M, Blank C, Schölmerich J, Hellerbrand C (2006). PD-L1 is induced in hepatocytes by viral infection and by interferon-α and -γ and mediates T cell apoptosis. J Hepatol.

[76] Gehring AJ, Sun D, Kennedy PT, Nolte-'t Hoen E, Lim SG, Wasser S, Selden C, Maini MK, Davis DM, Nassal M, Bertoletti A (2007). The level of viral antigen presented by hepatocytes influences CD8 T-cell function. J Virol.

[77] Kim JV, Kang SS, Dustin ML, McGavern DB (2009). Myelomonocytic cell recruitment causes fatal CNS vascular injury during acute viral meningitis. Nature.

[78] Sitia G, Isogawa M, Kakimi K, Wieland SF, Chisari FV, Guidotti LG (2002). Depletion of neutrophils blocks the recruitment of antigen-nonspecific cells into the liver without affecting the antiviral activity of hepatitis B virus-specific cytotoxic T lymphocytes. Proc Natl Acad Sci USA.

[79] Sitia G, Isogawa M, Iannacone M, Campbell IL, Chisari FV, Guidotti LG (2004). MMPs are required for recruitment of antigen-non specific mononuclear cells into the liver by CTLs. J Clin Invest.

[80] Iannacone M, Sitia G, Isogawa M, Marchese P, Castro MG, Lowenstein PR, Chisari FV, Ruggeri ZM, Guidotti LG (2005). Platelets mediate cytotoxic T lymphocyte-induced liver damage. Nat Med.

